# A Performance Optimized CSTBT with Low Switching Loss

**DOI:** 10.3390/mi14051039

**Published:** 2023-05-12

**Authors:** Hang Xu, Tianyang Feng, Wenrong Cui, Yafen Yang, David Wei Zhang

**Affiliations:** School of Microelectronics, Fudan University, Shanghai 200433, China

**Keywords:** CSTBT, shield gate, DC bias, low switching loss, conduction loss

## Abstract

A novel Performance Optimized Carrier Stored Trench Gate Bipolar Transistor (CSTBT) with Low Switching Loss has been proposed. By applying a positive DC voltage to the shield gate, the carrier storage effect is enhanced, the hole blocking capability is improved and the conduction loss is reduced. The DC biased shield gate naturally forms inverse conduction channel to speed up turn-on period. Excess holes are conducted away from the device through the hole path to reduce turn-off loss (E_off_). In addition, other parameters including ON-state voltage (V_on_), blocking characteristic and short circuit performance are also improved. Simulation results demonstrate that our device exhibits a 35.1% and 35.9% decrease in E_off_ and turn-on loss (E_on_), respectively, in comparison with the conventional shield CSTBT (C_on_-SGCSTBT). Additionally, our device achieves a short-circuit duration time that is 2.48 times longer. In high-frequency switching applications, device power loss can be reduced by 35%. It should be noted that the additional DC voltage bias is equivalent to the output voltage of the driving circuit, enabling an effective and feasible approach towards high-performance power electronics applications.

## 1. Introduction

Insulated Gate Bipolar Transistor (IGBT) is the core device in power electronic devices used for electric energy conversion and circuit control [1,2,3]. Various IGBT structures and techniques have been proposed and studied, including the field stop IGBT (FS-IGBT) [4], injection enhanced gate transistor (IEGT) [5] and carrier stored trench-gate bipolar transistor (CSTBT) [6]. With IGBT discrete devices and IGBT modules having an output power of up to 10 MW, they are widely utilized in high-voltage systems of Electric Vehicle, such as the main drive inverter, on-board charger (OBC), battery management system, on-board air conditioning control system, steering and other high-voltage auxiliary systems. In addition, IGBT is extensively employed in DC and AC charging piles, making them the cornerstone of the Electric Vehicle ecosystem.

For modern electronic systems represented by Electric Vehicles, higher operating frequency and higher power output are essential design goals. In the power converter system of Electric Vehicles (e.g., DC/DC converter, DC-to-DC inverter and the electronic power transformer), increasing the operating frequency can significantly benefit the system by reducing its volume, eliminating total harmonic distortion and improving power quality [7,8].

However, IGBT is still bottlenecked by technical and physical limitations, such as the unique trailing current and resulting switching loss that restrict the switching frequency to below 20 kHz [9,10,11]. Among the IGBTs mentioned above, CSTBT stands out as a promising candidate for the next-generation power electronics due to its ability to significantly reduce the on-state voltage while improving the carrier distribution in trench IGBT [12,13,14].

In a typical CSTBT, an additional carrier storage (CS) layer with a higher doping concentration is located between the P-type well region (P-body) and the N-drift region. In the on-state, the CS layer acts as a higher potential barrier that blocks holes from being collected by the emitter, leading to hole accumulation in the drift region near emitter side [15,16]. Accordingly, this causes a considerable number of electrons to be injected through the emitter, maintaining electrical neutrality and increasing the carrier concentration [17,18]. 

The switching frequency of optimized IGBTs is limited to 20 kHz to 40 kHz, which falls short of the desired 100 kHz, resulting in a tradeoff between high power and high frequency [19,20,21]. 

Therefore, improving the switching performance of IGBT has become a highly concerned issue, with two primary limiting factors listed below:

1. Inherent tail current affecting the switching performance.
2. Overheating caused by excessive switching loss, which is the main limiting factor at present [22].

Switching frequency refers to the number of times the IGBT is switched on and off within a second. Under the specified bus voltage and current, IGBT will generate power loss every time it switches. As the switching frequency increases, so does the power loss and temperature rise, which could lead to IGBT failure if the temperature exceeds the upper limit. The total power losses of an IGBT chip are typically composed of turn-on loss (E_on_), turn-off loss (E_off_) and conduction loss, and are proportional to the product of frequency and switching loss (E_off_ + E_on_).

**Figure 1:**
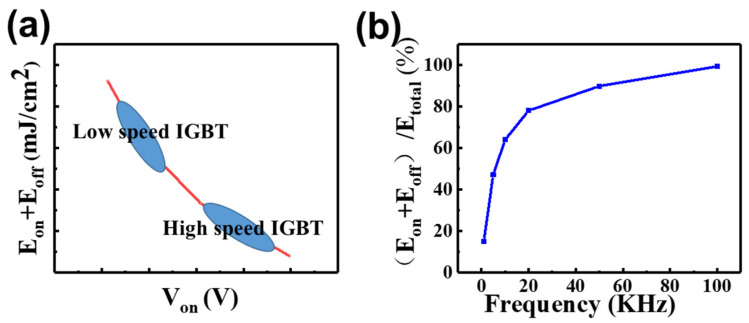
(**a**) Tradeoff relationship between V_on_ − (E_off_ + E_on_). (**b**) The proportion of E_off_ + E_on_ in total power loss under different frequency (Calculated according to IGBT data sheet).

In high frequency switching applications above 20 kHz, the reduction of switching loss becomes crucial, as it accounts for the majority of the total power consumption (above 60%), as shown in Figure 1b. In order to reduce the total power consumption under the switching frequency of 20~50 kHz, IGBT chip performance must be set at the position of high V_on_, low E_on_ and E_off_, like High speed IGBT in Figure 1a.

Currently, shield gate technology (SGT) is a promising way to reduce switching loss by decreasing Q_g_. However, the grounded bottom electrode in SGT increases channel resistance, leading to a serious impact on V_on_ and a corresponding rise in conduction loss. 

In this work, we propose a CSTBT device with a forward DC bias shield gate and hole path (DC-CSTBT). The characteristics of low V_on_, low E_on_, low E_off_ and high breakdown voltage are demonstrated simultaneously. The proposed device only made minor changes on the basis of conventional CSTBT and offers a simple and easy-to-implement structure and process. 

## 2. Structure and Process Flow

**Figure 2:**
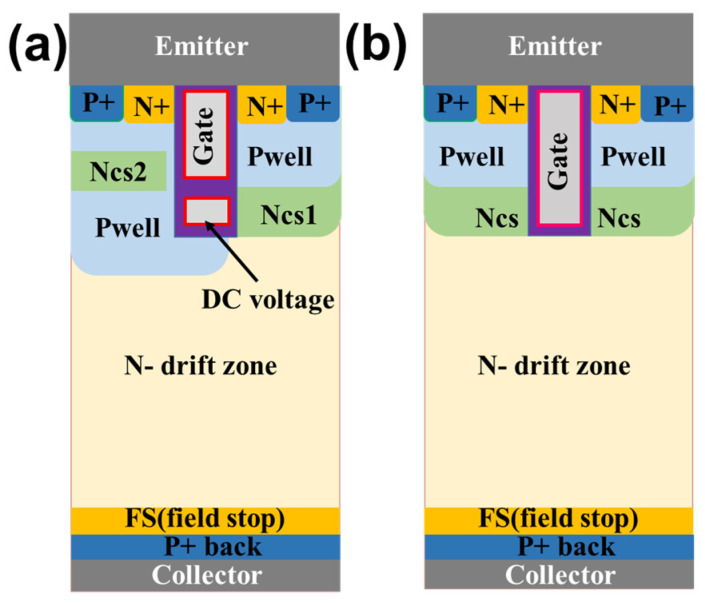
(**a**) The proposed DC-CSTBT; (**b**) Conventional CSTBT.

The schematic diagram of the proposed DC-CSTBT is shown in Figure 2a. Compared with the conventional CSTBT shown in Figure 2b, both devices have the same doping profile except for an extra N-type carrier storage layer (CS2).

In the proposed DC-CSTBT, the trench gate is split into two parts, including the upper and deeper parts. The upper part is connected to the gate control signal, controlling the turning on and off of the device. The deeper shield trench gate is connected to a positive DC bias. Generally, the DC bias value is the same as the gate saturation turn-on voltage, and there is no need to add the additional output of the driving circuit.

The CS2 region on the left is achieved by additional lithography and ion implantation. There is a gap between the trench and the CS2, and the state (on/off) of this hole path is completely controlled by the trench gate voltage. In the on state, the hole channel is closed, and the gap is inverted and acts as a whole CS storage layer with a CS2 layer. As the CS2 locates away from the bottom of the trench, its doping concentration is high to reduce V_on_ without blocking characteristic deterioration. Table 1 shows the major structural parameters of the proposed device.

**Figure 3:**
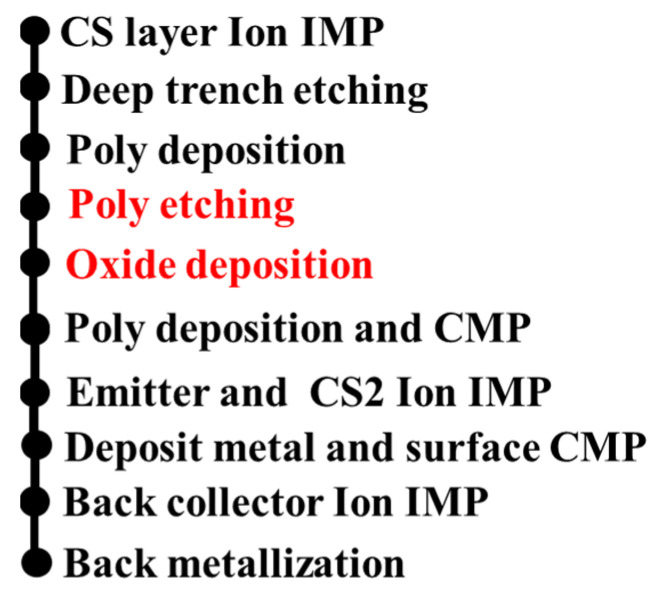
Process flow diagram. IMP (Ion implantation) CMP (Chemical mechanical polishing).Two additional processes are highlighted in red.

The split trench gate technology is compatible with the current manufacturing process. The process flow of the device is shown in Figure 3. The main process steps include epitaxial layer formation, ion implantation, trench etching, polysilicon deposition and etching, oxide layer formation, etc. Two additional processes compared with the conventional CSTBT process are highlighted in red, namely the polysilicon etching and the oxide layer deposition.

**Figure 4:**
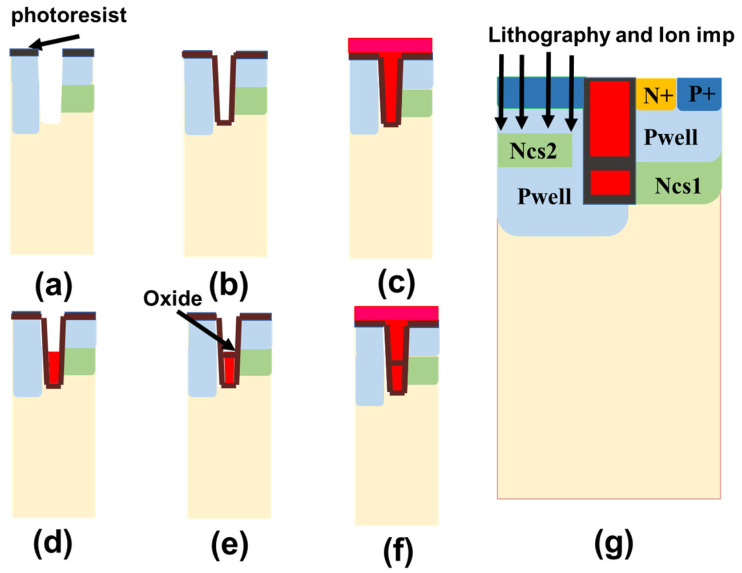
The formation process of DC bias shielding gate and extra CS2 in the proposed device. (**a**) deep trench etching. (**b**) oxide deposition. (**c**) polysilicon deposition. (**d**) polysilicon etching. (**e**) oxide deposition to complete the isolation. (**f**) polysilicon deposition to form the split gate. (**g**) the formation of CS2.

In addition, the detailed device process of split gate formation is shown in Figure 4a–f below. After deep trench etching (Figure 4a), an oxide (Figure 4b) and a very thick polysilicon (Figure 4c) is deposited in the trench. Then, part of the polysilicon is etched (Figure 4d), and only the bottom part as a shield gate is left. Subsequently, an oxide layer is deposited to complete the isolation of the upper and lower split gates (Figure 4e). Finally, polysilicon is deposited into the trench to form the split gate (Figure 4f). The split gate at the bottom serves as the DC bias shielding gate in the paper. Figure 4g shows the formation of CS2 on the left. A high-energy ion injection is added after an emitter injection, and the distribution of the CS2 layer is defined by mask.

Despite the slight increase in device fabrication complexity or cost associated with the proposed DC bias shielding approach, the process steps are completely consistent with those of existing trench IGBT devices. Additionally, the inclusion of a DC bias shield gate necessitates only minimal alterations to the driving circuit and requires the addition of only one lithography mask. This device has a simple structure and low cost, with great potential for practical applications. 

## 3. Simulation Results and Discussion

The device model is established by the Sentaurus Technology Computer-Aided Design (TCAD) simulation, and the process conditions are based on the SMIC 0.18 µm process node. The following four aspects of optimization brought by DC bias shielding are introduced. The structures contrasted below include a conventional CSTBT, a conventional shield CSTBT with a grounded shield gate (C_on_-SGCSTBT), DC-CSTBT, referring to CSTBT without a split gate, a split gate CSTBT with grounded shield gate and the proposed DC bias CSTBT, respectively.

A. Low E_on_

There are two factors contributing to the low E_on_. Firstly, the SGT gate structure reduces the Miller capacitance, which helps to decrease the E_on_. The Miller effect induces the formation of a platform voltage when driving the trench gate, which subsequently prolongs switching time and heightens switching loss. These consequences can have profoundly detrimental effects on the normal operation of the IGBT. Secondly, the shield gate DC bias technology accelerates the formation of conductive channels, further reducing the E_on_.

(1) low Q_GC_

**Figure 5:**
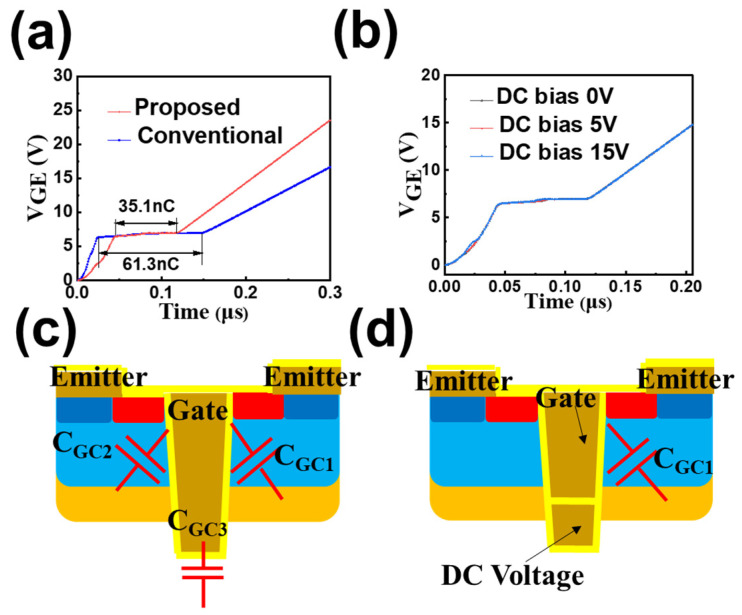
(**a**) Gate charge curve for conventional CSTBT and the proposed DC-CSTBT. (**b**) Gate charge curve of the proposed device under different DC bias. (**c**) Main distribution of C_GC_ in conventional device. (**d**) Main distribution of C_GC_ in the proposed device.

In the conventional CSTBT structure, the whole C_GC_ is composed of three parts, as depicted in Figure 5c; namely, C_GC1_, C_GC2_ and C_GC3_. C_GC1_ and C_GC2_ are mainly related to gate oxide thickness and the coupling area between trench and collector. Among these three parts, C_CG3_ is the most dominant factor. Compared with a conventional CSTBT, the deeper trench of the proposed device is DC biased (equivalent to AC ground), resulting in a smaller coupling area between the gate and collector. The charging time of the Miller capacitor is reduced by 42.7% in the proposed DC-CSTBT compared with the conventional CSTBT. Q_GC_ is reduced from 61.3 nC to 35.1 nC, and the Miller capacitance of the proposed device, equivalently, is decreased from 8.636 nF/cm^2^ to 2.57 nF/cm^2^. The reduction in Miller capacitance is mainly transformed into C_GE_. The simulation results indicate that the Q_GC_ of the proposed device remains constant under a different DC bias, as depicted in Figure 5b. Therefore, a higher DC bias can enhance the carrier storage effect without deteriorating the Miller effect.

(2) Faster turn-on by forward biased bottom inversion channel

C_on_-SGCSTBT will bring the problem of excessive V_on_. To address this, the improved DC-CSTBT is proposed here.

**Figure 6:**
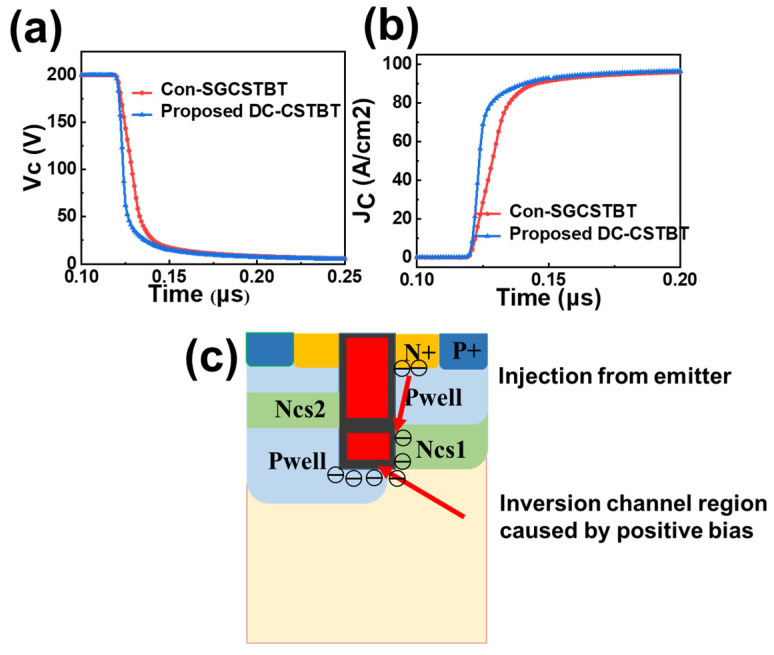
Turn-on curve (**a**) V_c_ curve during turn-on process; (**b**) J_c_ curve during turn-on process; (**c**) Working mechanism of DC voltage bias shield gate.

Figure 6 shows the turn-on curve of the proposed device under a clamped load. The collector current voltage and collector current of the proposed device is swifter than C_on_-SGCSTBT, as depicted in Figure 6a,b. This improvement can be attributed to the inverse channel forward formed by the DC voltage at the trench bottom rather than the grounded deep trench. The formation of a conductive channel is accelerated and the turn-on process is sped up effectively by the DC biasing technique. 

The turn-on process of the IGBT is as follows. First, a positive bias is applied at the trench, which inverts the P-base region to form an N channel. Then, the emitter releases electrons into the drift region through this channel, leading to the potential drop in the drift region. When the potential is low to a certain threshold, the collector PN junction conducts forward, and holes are injected into the N-region; conductance modulation effect starts.

**Figure 7:**
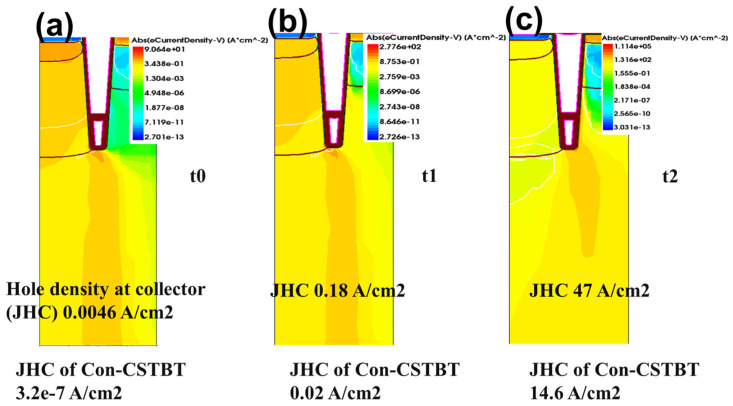
Electron current distribution during turn-on process of the proposed DC-CSTBT with 15 V DC bias (**a**) at t_0_; (**b**) at t_1_; (**c**) at t_2_; (t_0_ t_1_ t_2_ three moments are after turn-on at 1 × 10^−7^ ns, 2 × 10^−7^ ns, 3 × 10^−7^ ns, respectively).

Figure 7 shows the distribution of electron current during the turn-on state. Large electron currents flowing to the drift region can be clearly seen at the trench bottom. The mechanism can be explained as follows. There exists a DC bias at the shield gate and a corresponding electric field pointing from the trench bottom to the emitter. Consequently, electrons in the emitter are injected through the inversion layer around the shield gate, resulting in a decrease in the N-drift potential.

Moreover, the hole current density at the collector region is marked, which can further compare the collector injection speed, as illustrated in Figure 7a–c. At t_0_, the hole current density at the collector (J_CH_) of DC-CSTBT is 10^4^ larger than that of the normal grounded shield gate. At t_1_, J_CH_ of DC-CSTBT is also nine times larger. Until t_2_, both reach an order of magnitude of 47 A/cm^2^ and 14.6 A/cm^2^, respectively. It is further proved that the DC bias shield can speed up the device turn-on process. 

**Figure 8:**
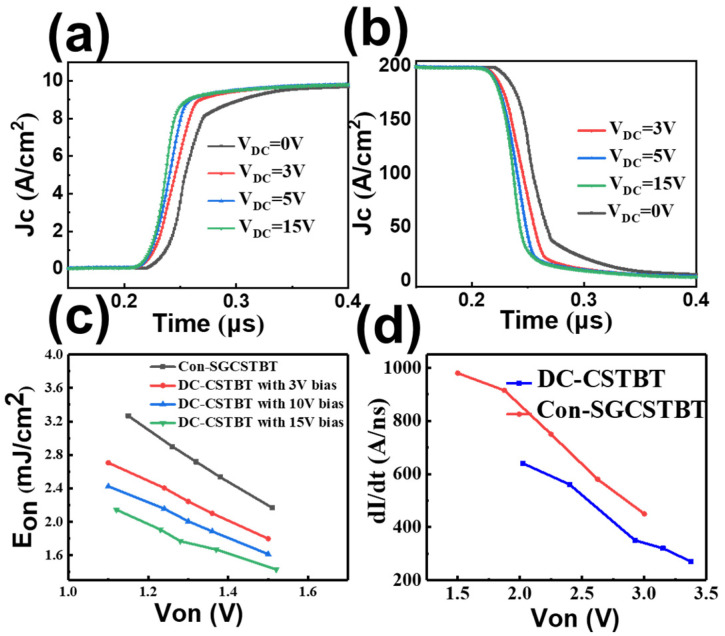
(**a**) J_c_ curve during turn-on process under different DC bias; (**b**) V_c_ curve during turn-on process; (**c**) Trade off relationship between E_on_ and V_on_ in the proposed DC-CSTBT and C_on_-SGCSTBT. (**d**) V_on_ versus dI/dt of the proposed DC-CSTBT and C_on_-SGCSTBT in turn-on process.

Figure 8 shows the turn-on curve of the proposed DC-CSTBT (under various DC bias). It can be seen that the J_C_ of the DC-CSTBT with a higher DC voltage is much swifter. A higher DC bias contributes to a stronger electric field to promote the incident of electrons. In addition, during the turn-on process, dV/dt and dI/dt are significantly improved, and the turn-on loss can be reduced. The simulation results show that, compared with the C_on_-SGCSTBT, the E_on_ of the proposed device can be reduced by 35.9%, from 3.2 mJ/cm^2^ to 2.05 mJ/cm^2^.

B. Low E_off_

**Figure 9:**
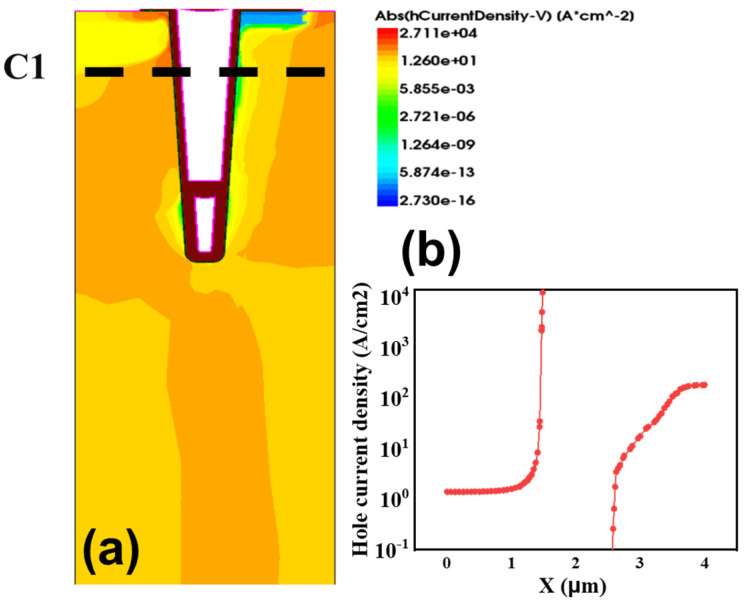
Transient hole current distribution during turn-off (**a**) curve during turn-off process at t_1_ (a period of time after the turn off time t_0_); (**b**) hole current value along C1 at t_1_.

Figure 9a illustrates the transient hole current distribution during the turn-off at t_1_ (a period of time after the turn on time t_0_) of the DC-CSTBT and hole current values along C_1_. It can be seen that the highest hole current is concentrated at the designed hole path, reaching more than 1 × 10^4^ A/cm^2^ as depicted in Figure 9b. 

In the turn-off process, the inversed hole path recovers into the P-region with V_G_ transferring to zero. Consequently, the accumulated holes in the drift region are swept out through the inversion layer around the hole path region, accelerating hole extraction to make the drift zone reach neutrality as soon as possible.

**Figure 10:**
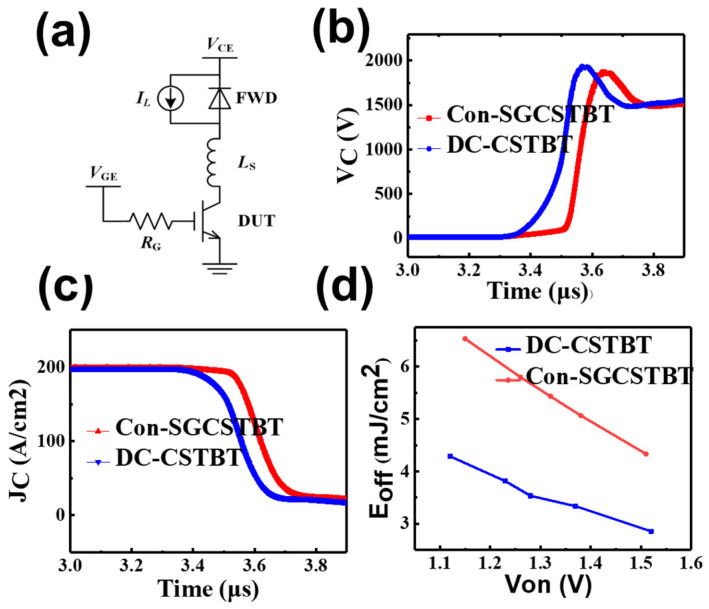
(**a**) Circuit schematic for turn-off transient analysis. (V_cc_ = 1750 *V* L_c_ = 1 mH L_s_ = 1.5 fH R_g_ = 10 ῼ). (**b**) Turn off waveform of collector voltage. (**c**) Turn off waveform of collector current. (**d**) Trade off relationship between E_off_ and V_on_ in the proposed DC-CSTBT and C_on_-SGCSTBT.

The mixed-mode, clamped, inductive switching simulation is performed to study the turn-off performance. The simulated devices are set to the same current of 10 A at the current density of 100 A/cm^2^. The DC bus voltage (V_bus_) is set to 1500 V. The load inductance L_c_ load is 1 mH and the circuit is shown in Figure 10a. The gate voltage decreases from 15 to 0 V to turn off the device. The simulated turn-off process of C_on_-SGCSTBT and the proposed DC-CSTBT (with same V_on_ of 1.5 V) are shown in Figure 10b,c. Clearly, the T_off_ of the DC-CSTBT is much smaller than that of C_on_-SGCSTBT, which indicates that the E_off_ of the proposed device is more excellent.

Figure 10d illustrates the relationship between E_off_ and V_on_ for the two different structures, after changing the peak P-collector doping concentration. Under V_on_ = 1.15 V, the E_off_ of the proposed DC-CSTBT is 6.5 mJ/cm^2^, which is 36.1% lower than that of the C_on_-SGCSTBT (4.15 mJ/cm^2^). In addition, the switching loss, including E_on_ and E_off_, are further compared, as shown below. 

**Figure 11:**
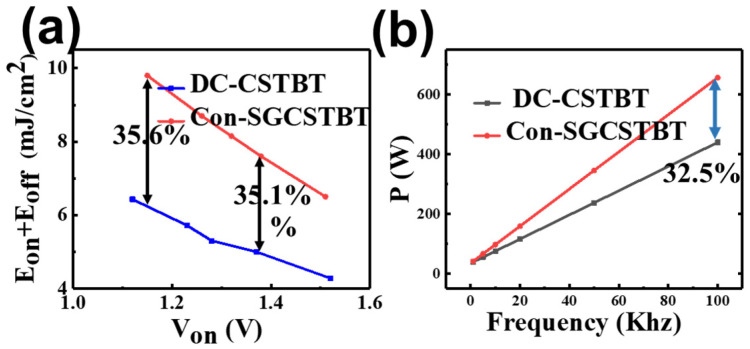
(**a**) Trade off relationship between V_on_ − (E_off_ + E_on_). (**b**) Relationship between power loss and switching frequency.

Figure 11 shows the tradeoff curves of V_on_ − (E_off_ + E_on_) for the devices. It is observed that the tradeoff curves of DC-CSTBT is obviously more optimized than C_on_-SGCSTBT. This is mainly due to the presence of both a hole path and emitter electron injection acceleration techniques, which improve turn-on and turn-off, respectively. For the same V_on_ 1.38 V, E_off_ + E_on_ of DC-CSTBT is decreased by 35.1% compared with the C_on_-SGCSTBT. However, with the V_on_ of 1.14 V, this reduction percentage is still higher. Considering the same E_off_ + E_on_ of 6.4 mJ/cm^2^, the V_on_ of the DC-CSTBT can be 25.8% lower than that of the C_on_-SGCSTBT. Therefore, DC-CSTBT have a better tradeoff between V_on_ and E_off_ + E_on_ than C_on_-SGCSTBT. Correspondingly, the calculated power loss in high-frequency switching applications (100 kHz) can be reduced by 32.5% as shown in Figure 11b.

C. Blocking characteristics

As mentioned above, the breakdown voltage of the trench IGBT is affected by the deep trench structure. The breakdown voltage of the trench IGBT is lower than that of the traditional planar type because of the pre-breakdown of trench bottom.

**Figure 12:**
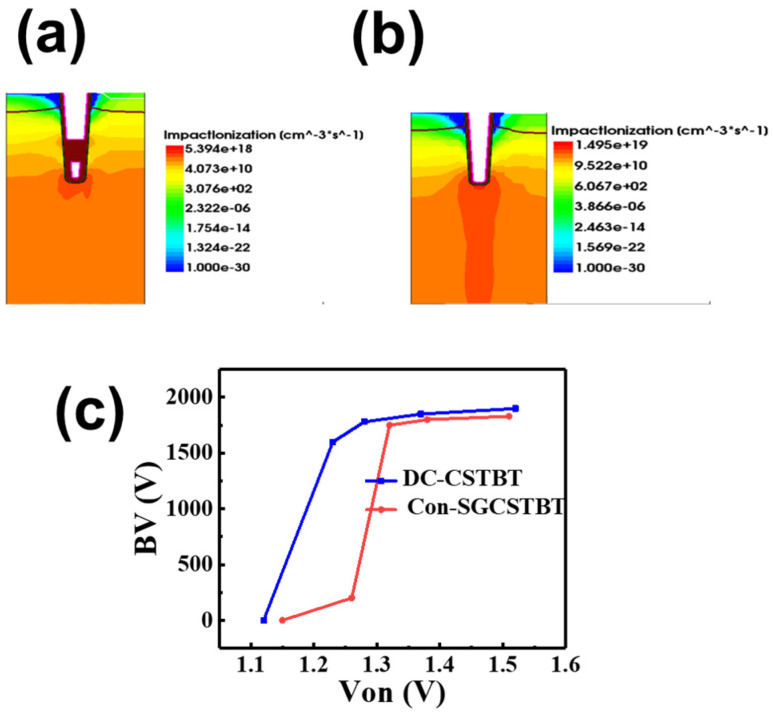
(**a**) Impact ionization rate of DC-CSTBT under 1800 V reverse bias. (**b**) Impact ionization rate of conventional CSTBT under 1800 V reverse bias. (**c**) Dependence of the breakdown voltage and varying V_on_.

Especially with the introduction of the carrier enhancement layer, the electric field at the bottom becomes too concentrated. The reason for the high electric field is the largest potential difference at the trench bottom. The electric potential of the trench is 0 V, which is the lowest potential at the same depth in the drift region. Breakdown initially occurs at the bottom of the trench. Some structures have previously been proposed to attenuate the overly concentrated electric field, such as implanting the P-type doped well at the bottom. However, the conduction loss will increase significantly because of the drift zone resistance increase. To address this issue, the proposed DC bias device employs a deeper trench electrode connected with a forward DC bias to reduce the potential difference at the bottom trench by raising the potential at the shield gate, thus relieving the electric field at the bottom of the trench effectively. Figure 12a,b show the impact ionization distribution under same reverse bias at 1800 V. It should be noted that C_on_-SGCSTBT shows similar breakdown characteristics as a conventional CSTBT for the same electric field distribution at the trench bottom, while the proposed device can reduce the peak impact by about 50% compared with C_on_-SGCSTBT. 

Figure 12c illustrates the tradeoff relationship between BV and V_on_ of C_on_-SGCSTBT and the proposed DC-CSTBT (by changing the doping concentration of the CS layer). Under the same V_on_, the proposed device always has a higher BV because of the weakened electric field at the trench bottom. For C_on_-SGCSTBT, the BV starts to decrease when V_on_ is smaller than 1.35 V (reduce the conduction pressure drop by increasing the concentration of CS). While the proposed device can maintain 1600 V withstand voltage until V_on_ is smaller than 1.2 V. The present device achieves a better tradeoff relationship.

D. Conduction characteristics

**Figure 13:**
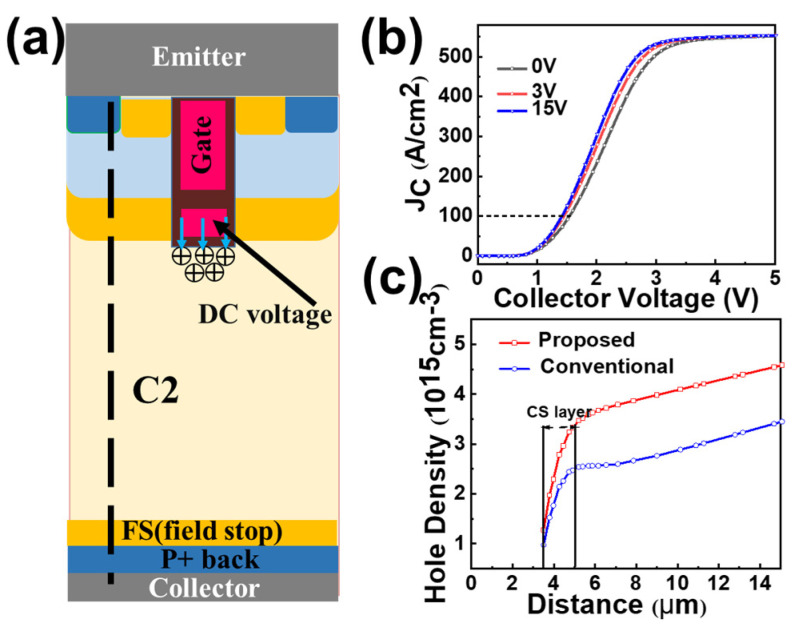
(**a**) Schematic diagram of carrier storage effect enhancement mechanism. (**b**) I_D_–V_D_ curves of DC-CSTBT under different DC bias. (**c**) Distributions of the hole carrier density along line C2 shown in at collector current density J = 100 A/cm^2^.

The forward DC bias at the deep shield trench repels positively charged holes because of mutual repulsion between positive charges, promoting the accumulation of holes near the trench bottom, as depicted in Figure 13a. Therefore, a large number of holes accumulates at the emitter side. In order to maintain electrical neutrality, the emitter should emit a corresponding quantity of electrons. With the increase in carrier doping concentrations in the drift region, the on-resistance of the device is reduced. Figure 13b shows the comparison of output characteristics of the proposed device under different forward DC voltages including 0 V, 3 V and 15 V. It is clear that V_on_ decreases with DC bias rises, as a higher DC voltage contributes to a stronger hole block effect. Figure 13c compares the distributions of the hole carrier density along line C2 for DC-CSTBT and with a DC bias at collector current density J = 100 A/cm^2^. As the DC bias at the deeper electrode only contributes to a higher hole carrier density near the emitter, only a little penalty in the switching losses will be added.

E. Other characteristics

Output characteristics, transfer characteristics and short circuit characteristics are also studied. Simulation results show that the J_C_–V_G_ curve does not change, while the saturation current and short circuit characteristics are greatly optimized.

**Figure 14:**
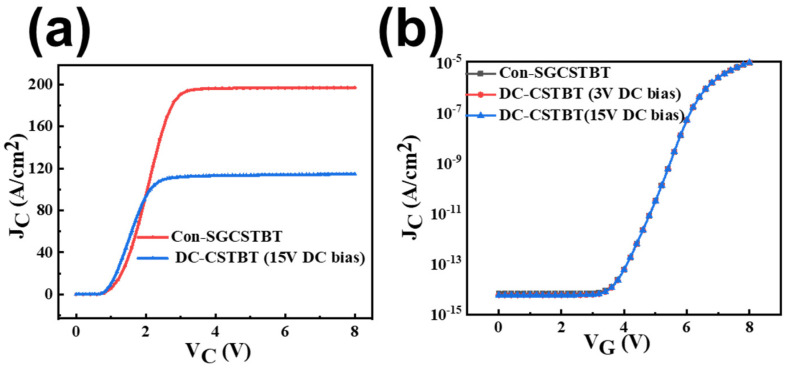
(**a**) Output characteristics of C_on_-SGCSTBT and DC-CSTBT. (**b**) Transfer characteristics of C_on_-SGCSTBT and DC-CSTBT.

Figure 14a shows that the saturation current density is significantly reduced compared with C_on_-SGCSTBT, since no current flows through the left half of the proposed DC-CSTBT. In addition, Figure 14b shows the transfer characteristics (J_C_–V_G_ curve) of C_on_-SGCSTBT and the proposed device under the same V_on_. The curve of both devices almost overlap, indicating that the proposed structure only optimizes the device characteristics by adding a DC bias shield structure, while preserving the basic transfer characteristics of the device.

**Figure 15:**
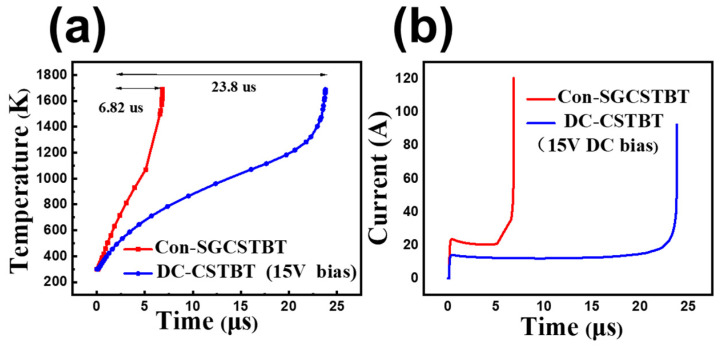
(**a**) Maximum device temperature curves in the short-circuit situation of the two devices. The self-heating effect is considered by the LAT.TEMP model with the initial device temperature of 300 K. (**b**) Simulated short-circuit current of the C_on_-SGCSTBT and the proposed DC-CSTBT devices.

Figure 15 shows short-circuit waves. It indicates that the DC-CSTBT withstands a significantly longer short-circuit duration time (device failure time is defined when lattice temperature reaches 1687 K) than that of the C_on_-SGCSTBT before failure. Owing to the smaller saturation current density, the short-circuit time of the proposed DC-CSTBT structure is about 2.48 times longer than that of the C_on_-SGCSTBT.

## 4. Conclusions

A novel DC-CSTBT with additional forward DC bias is proposed and investigated by numerical simulations. The trench is split into two parts, the upper one serves as a normal gate electrode while the deeper one is biased by forward DC voltage to block holes from flowing to the emitter. By combining the enhanced carrier storage effect caused by forward bias and hole path, the proposed device shows excellent performance. Specifically, the new structure exhibits a 42.7% decrease in Q_GC_; E_off_ and E_on_ are reduced by 35.1% and 35.9%. Importantly, the process steps are completely consistent with the existing trench IGBT devices. Only two simple process steps are added, and only one additional lithography mask is needed, making the device highly practical with the potential for low cost and a simple structure.

## Figures and Tables

**Table 1:** Major structural parameters.

Parameter	Value
Cell pitch	4 μm
Gate oxide thickness	120 nm
N-drift doping	5.9 × 10^13^ cm^−3^
CS doping	4.0 × 10^14^ cm^−3^
High doping CS (CS2)	1.5 × 10^17^ cm^−3^
P-well doping	3.0 × 10^16^ cm^−3^
Trench depth	5 μm

## Data Availability

Not applicable.
